# A geospatially resolved wetland vulnerability index: Synthesis of physical drivers

**DOI:** 10.1371/journal.pone.0228504

**Published:** 2020-01-30

**Authors:** Zafer Defne, Alfredo L. Aretxabaleta, Neil K. Ganju, Tarandeep S. Kalra, Daniel K. Jones, Kathryn E. L. Smith

**Affiliations:** 1 Woods Hole Coastal and Marine Science Center, U.S. Geological Survey, Woods Hole, MA, United States of America; 2 Integrated Statistics, U.S. Geological Survey, Woods Hole, MA, United States of America; 3 Utah Water Science Center, U.S. Geological Survey, Salt Lake City, UT, United States of America; 4 St. Petersburg Coastal and Marine Science Center, U.S. Geological Survey, St. Petersburg, FL, United States of America; China University of Geosciences, CHINA

## Abstract

Assessing wetland vulnerability to chronic and episodic physical drivers is fundamental for establishing restoration priorities. We synthesized multiple data sets from E.B. Forsythe National Wildlife Refuge, New Jersey, to establish a wetland vulnerability metric that integrates a range of physical processes, anthropogenic impact and physical/biophysical features. The geospatial data are based on aerial imagery, remote sensing, regulatory information, and hydrodynamic modeling; and include elevation, tidal range, unvegetated to vegetated marsh ratio (UVVR), shoreline erosion, potential exposure to contaminants, residence time, marsh condition change, change in salinity, salinity exposure and sediment concentration. First, we delineated the wetland complex into individual marsh units based on surface contours, and then defined a wetland vulnerability index that combined contributions from all parameters. We applied principal component and cluster analyses to explore the interrelations between the data layers, and separate regions that exhibited common characteristics. Our analysis shows that the spatial variation of vulnerability in this domain cannot be explained satisfactorily by a smaller subset of the variables. The most influential factor on the vulnerability index was the combined effect of elevation, tide range, residence time, and UVVR. Tide range and residence time had the highest correlation, and similar bay-wide spatial variation. Some variables (e.g., shoreline erosion) had no significant correlation with the rest of the variables. The aggregated index based on the complete dataset allows us to assess the overall state of a given marsh unit and quickly locate the most vulnerable units in a larger marsh complex. The application of geospatially complete datasets and consideration of chronic and episodic physical drivers represents an advance over traditional point-based methods for wetland assessment.

## Introduction

Coastal wetlands provide a multitude of ecosystem services, including habitat provision, recreational activities, coastal protection, and carbon sequestration. However, the stability of coastal wetlands is affected by processes that compromise their structural integrity. These processes include, but are not limited to, shoreline erosion, eutrophication, sediment supply [1–3], exposure to salinity changes [4], and other external forces. External forces include anthropogenic modification (e.g., urban encroachment, mosquito ditching), episodic events such as coastal storms [5, 6], climate change [7–9], and sea level rise [10–12]. Quantifying these processes may be based on field measurements, historical and modern geospatial data, and numerical modeling. Field measurements usually have higher accuracy and temporal coverage, but limited spatial extent. However, they can be used as ground truth data for geospatial datasets from remote sensing, which can cover much larger areas, and as calibration data for numerical model simulations, which can provide better spatiotemporal coverage. Geospatial datasets and numerical model solutions are therefore valuable resources for robust assessment of entire wetland systems, in comparison to point or transect-based methods. Geospatial datasets based on remote sensing and aerial imagery have already been widely been used to inventory and classify coastal wetlands [13, 14]

Considering the rate of vegetative cover loss as a sign of vulnerability, a group of indicators can be identified. For example, lower elevation marshes are more vulnerable to inundation under sea-level rise than higher elevation marshes. They are also more likely to be exposed to wave attack because of their proximity to shoreline [15]. Additionally, for parts of marshes adjacent to water, shoreline change can be used as an indicator of vegetated-land loss over time. Recently the unvegetated-vegetated marsh ratio (UVVR) has been proposed as an integrative metric of wetland vulnerability, as it correlates with net sediment budgets across a range of microtidal marshes [16]. The UVVR therefore links open-water conversion and sediment transport, and is a necessary quantity for estimating ecosystem services that are reliant on total vegetated marsh area (e.g., wave attenuation, carbon storage).

Eutrophication through excessive nitrogen loading destabilizes marsh vegetation by increasing above-ground biomass while decreasing below-ground biomass [1]. Increasing urbanization in the watersheds tends to intensify nutrient loads to the estuaries [17]. Estuaries with poor flushing and long residence times tend to retain nutrients within the system, leading to eutrophication and possibly destabilization of marsh vegetation.

Sediment supply contributes to the resiliency of salt marshes through vertical accretion [18]. Consequently, biomass production in salt marshes is positively correlated with mean tidal range, and therefore has the potential to increase vertical stability with respect to sea-level rise [19, 20]. Generally, a higher suspended sediment concentration (SSC) in the water column can be an indication of increased sediment availability, but associating it with the net sediment supply to the marsh system requires understanding the hydrodynamic setting and sediment transport mechanisms of the system [3]. Episodically, storm surges in micro- and meso-tidal regimes have been shown to be major sources of sediment [21].

Salinity tolerance by estuarine marsh vegetation varies between species. Generally, moving towards the wetland-upland interface soil salinity starts to decline. However, storm surge due to hurricanes may induce high salinity further inland. Studies have implicated elevated salinity and surge persistence as factors contributing to marsh dieback [22, 23].

Similarly, episodic releases of toxic contaminants during destructive events expose flora and fauna within the marsh to potentially deleterious impacts. These impacts may reduce the ability of marshes to provide suitable habitat or may result in the storage of such contaminants in marsh soils. The contaminant exposure was considered as potential vulnerability that can be triggered by episodic events such as coastal storms.

Each of these indicators explain a different aspect of vulnerability, has different units, scales and ranges, there confounding a simple vulnerability assessment. A straightforward solution is to normalize all external variables and aggregate them to form a single, dimensionless index. This comes with some loss of information, such as a high value of an aggregated index at two different locations within a system will not reveal which factors are leading to the elevated value [24–26]. Sometimes, even after normalization the distribution of values can be influenced by the formulation (e.g., step, asymptotic, exponential functions) [27]. Nevertheless, the use of a single index as vulnerability metric is practical for present-day assessment and may facilitate forecasting. For example, researchers developed a coastal vulnerability index as a metric of potential coastal response to changes such as sea level rise [28, 29]. For their evaluation, they first ranked and then aggregated a set of parameters that included tidal range, wave height, coastal slope, shoreline change, geomorphology, and historical rate of relative sea level rise. This index was then used in a Bayesian statistical approach to the provide future estimates of coastal vulnerability to sea level rise [30]. Another study used a combination of geospatial bio-geophysical data and climate model outputs to create and map indexes of coastal vulnerability in an urbanized coastal ecosystem for the past, current and future scenarios [31]. Other examples for the use of an aggregated index includes a submergence vulnerability index that assesses the vulnerability of coastal zones of Louisiana to submergence due to local sea level rise using the wetland elevation and hydrologic data [32]; a vulnerability index to study the coastal wetlands of Yangtze river estuary China to sea level rise [33]; and a single vulnerability index based on different biochemical parameters to study the role of vegetation in coastal wetland ecosystems [34]. More recently, multimetric indices have been used in various studies for integrated assessments of ecosystem conditions of coastal wetlands; such as evaluating salt marsh restoration success [35]; developing condition indices based on rapid assessment of coastal tidal wetlands in New England, USA; and Gulf of Mexico [36]; and assessing condition and detecting change at the salt marshes from five national parks along the northeastern coast of USA based on vegetation and nekton metrics [37]. Similarly, based on a conceptual ecological model, researchers also created salt marsh metrics to monitor for sustainable management of Northern Gulf of Mexico [38]. These studies depend on on-site point measurements and useful in assessment of specifically selected locations, but results can be extended to larger areas only if the measurements are considered to be representative at larger scales.

In this study, we synthesize geospatial data from numerous sources to establish a vulnerability index with spatially continuous coverage for coastal wetlands. The proposed aggregated index is based on an equal weighted combination of all the available variables and quantifies relative vulnerability within the given domain. We introduce a novel approach to divide a marsh complex in to hydrologically defined marsh units and summarize the index at each unit. The index is customizable according to the interest, and extensible allowing for inclusion of new indicators. To demonstrate this, we explore its dependency to different temporal scales for its dependency on chronic (persistent) and episodic (extreme) indicators, specifically associated with Hurricane Sandy, which made landfall near the study area in October 2012 [39]. Extreme storms are expected to induce salinity stress, lateral erosion, and enhanced sediment transport in estuary and wetland environments, and are therefore important to a vulnerability assessment. We then apply Principal Component Analysis (PCA) to explore the parameter space and provide weights for the relative contribution of each parameter to the vulnerability index. Additionally, we apply a clustering algorithm to separate the domain into a set of subregions that share common characteristics.

**Fig 1:**
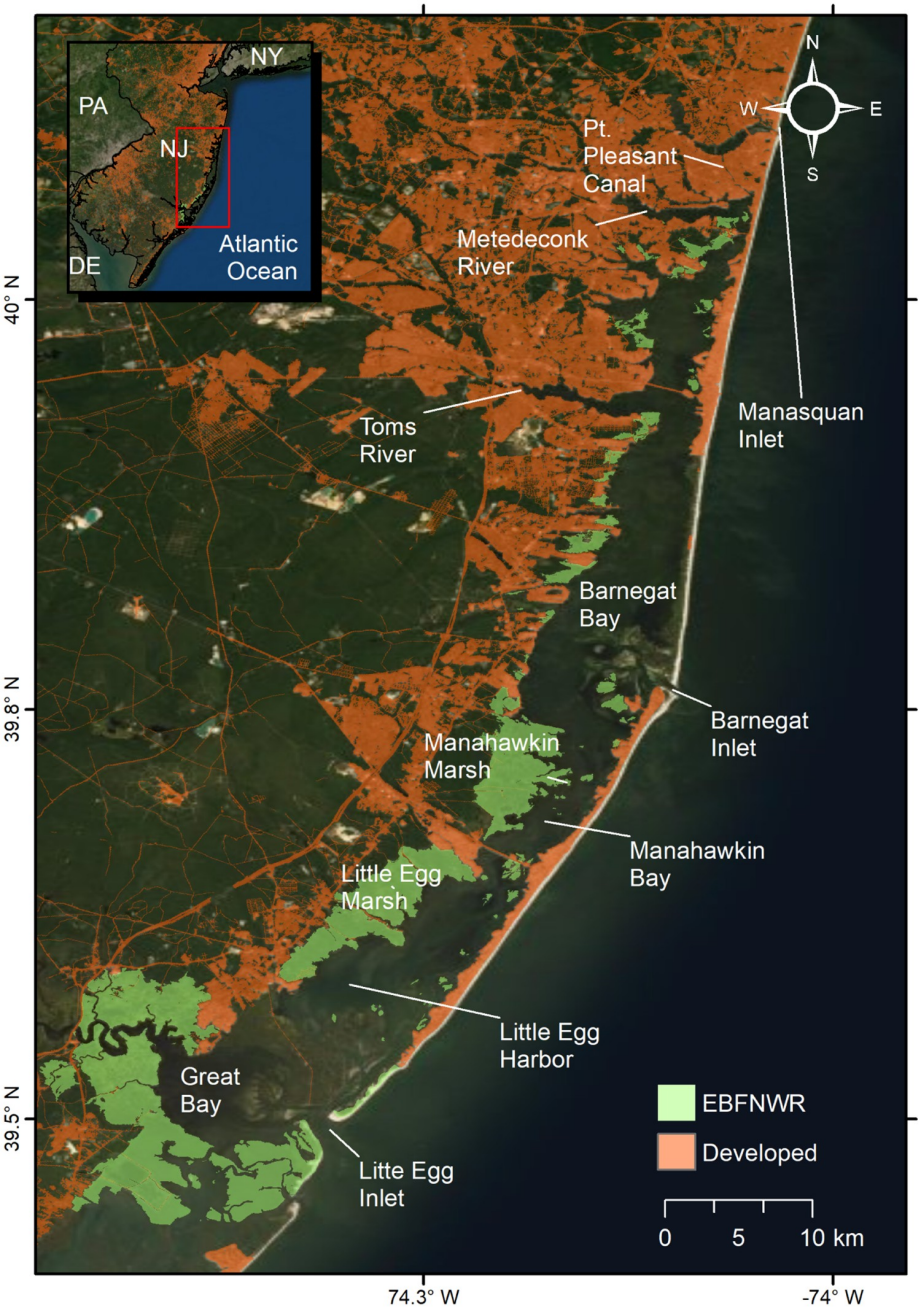
Overview map. E.B. Forsythe National Wildlife Refuge (EBFNWR) spans over Barnegat Bay, Little Egg Harbor, and Great Bay in New Jersey. The bay is connected to the ocean through Little Egg Inlet, Barnegat Inlet, and Pt. Pleasant Canal (via Manasquan Inlet in the north). Developed areas source: Multi-Resolution Land Characteristics Consortium’s National Land Cover Database 2011. World Imagery source: Esri, DigitalGlobe, GeoEye, Earthstar Geographics, CNES/Airbus DS, USDA, USGS, AeroGRID, IGN, and the GIS User Community.

We demonstrate the application of these approaches within the salt marsh complex of E.B. Forsythe National Wildlife Refuge (EBFNWR), which spans the Barnegat Bay-Little Egg Harbor (BBLEH) estuary and Great Bay in New Jersey, USA (Fig 1). The system has strong longitudinal spatial gradients from south to north: the watershed transitions from undeveloped to developed, tidal range decreases, and oceanic exchange decreases. Additionally, the northern bay has more freshwater input and a higher incidence of sediment bound contaminants [40–43].

## Data and methods

**Fig 2:**
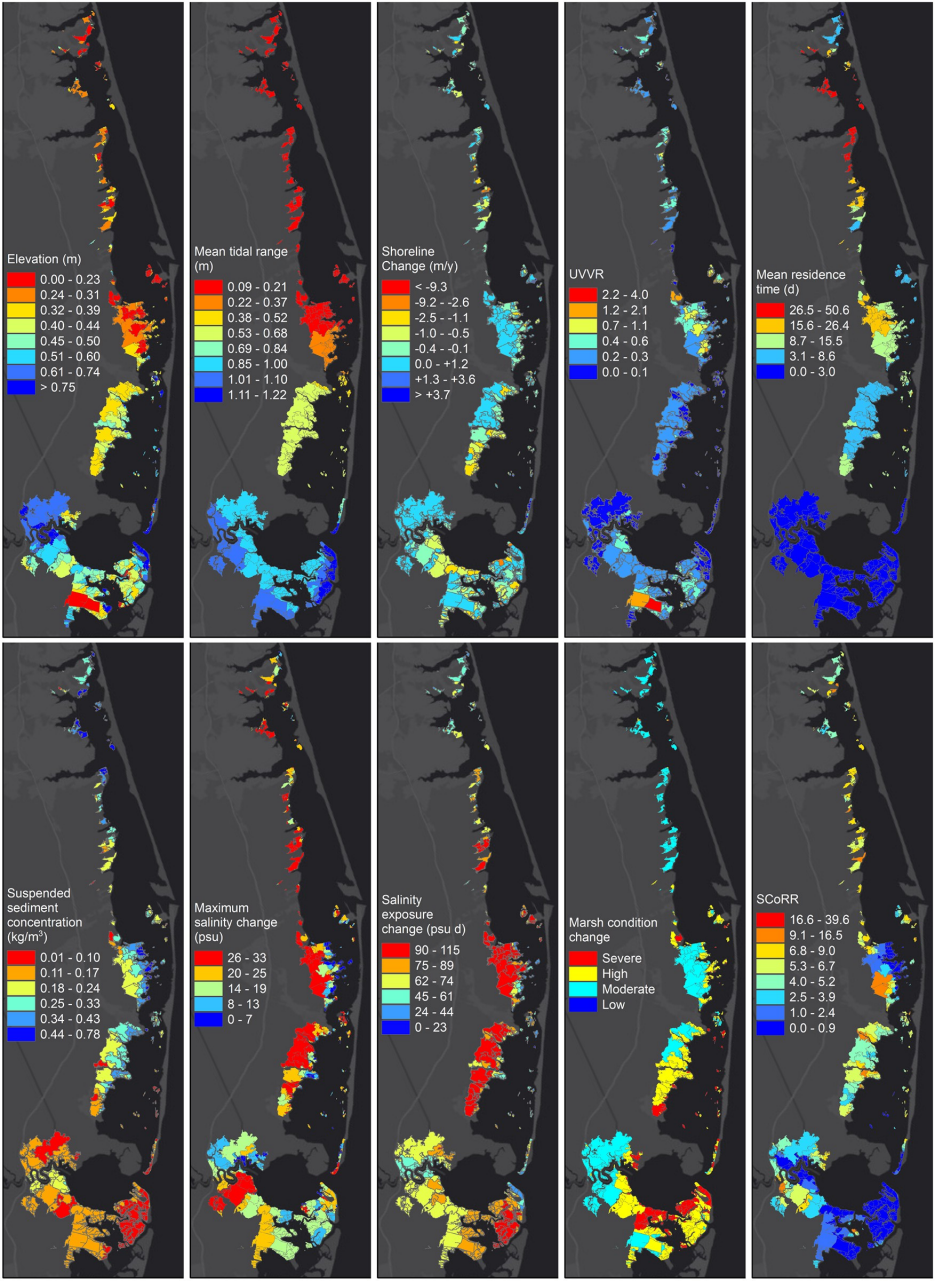
Wetland data layers. Underlying geospatial data has been averaged over the conceptual marsh units. Top row (from left to right): Elevation, mean tidal range, shoreline change, unvegetated to vegetated marsh ratio, and mean residence time. Bottom row: suspended sediment concentration, maximum salinity change, salinity exposure change, marsh condition change, and sediment-bound contaminant index.

The vulnerability index is based on various data sources including field observations, remote sensing, regulatory information and numerical models. first the underlying data has been summarized over the newly defined conceptual marsh units. Then the indicators were defined by rearranging the source data so that the larger values indicate higher vulnerability and were categorized as chronic or episodic indicators (Fig 2). The indicators were then ranked and aggregated to create a wetland vulnerability index. PCA, CA and hot spot analysis were used to further analyze the data. Marsh units definition and each source dataset are described in detail below and are available at https://www.sciencebase.gov/catalog/item/5b69d9b4e4b006a11f77597b.

### Conceptual marsh units

A union of wetland classification [44] and delineation of water bodies in emergent wetlands in coastal New Jersey [45] was used to define the domain boundaries. Conceptual marsh units were defined by geoprocessing of surface elevation raster data within the domain. Specifically, flow accumulation based on the relative elevation of each raster cell was used to determine the ridge lines that separated each marsh unit, while the surface slope was used to automatically assign each unit a drainage point, where water was expected to drain through. This procedure generally resulted in two types of marsh units: predominantly larger units inland, where drainage points were constrained by the topographic ridges, and predominantly smaller units near the marsh-estuary boundary, where there was a relatively stronger elevation gradient towards the edge. Because most of the smaller units were peripheral and not geographically relevant individually, these isolated units were merged with the nearest adjacent unit iteratively until a minimum surface area of 5,000 *m*^2^ was achieved. If a cluster of isolated units were not adjacent to a larger unit, they were aggregated to create a new unit [46].

### Chronic indicators

Chronic indicators include data layers that describe the geospatial boundaries of the data (e.g., elevation and shorelines) or define a characteristic delineation of vegetation (e.g., ratio of unvegetated to vegetated area in a marsh unit). These are typically variables that are modified by annual-to-decadal scale processes, and do not vary over tidal-to-seasonal timescales. In some cases, specifically tide range and residence time, these are external variables that act on the marsh system.

- **Lower elevation (ELEVA)**: The elevation data are derived from the 1/9 arc-second resolution U.S. Geological Survey National Elevation Data (USGS NED). Geological Survey National Elevation Data (USGS NED) at 1/9 arcsecond resolution. These elevation data were resampled to 1 meter and a mean elevation for each marsh unit was computed. [47].
- **Lower tidal range (TIDER)**: Mean tidal range was calculated as the difference in height between mean high water (MHW) and mean low water (MLW) using the VDatum (v3.5) software (http://vdatum.noaa.gov/). The values were interpolated over a 0.0003° (∼30 m) resolution grid and extended to the entire marsh domain with a nearest neighborhood method. These were then averaged over each marsh unit [48].
- **Shoreline change rate (SHORE)**: Evolution of shoreline position is determined by the balance between erosion and accretion along the coast. Shoreline change rates calculated from digital vector shorelines acquired from historic topographic sheets, aerial photography, and/or lidar [49], were averaged along the shoreline of each salt marsh unit to generate this layer.
- **Higher unvegetated to vegetated ratio (UVVR)**: The ratio of unvegetated area to vegetated area was calculated from wetland map code delineation [44].
- **Higher residence time (RESID)**: The residence time data layer was derived using particle tracking from a 7-month hydrodynamic simulation [50, 51], and projected on the marsh units [52].

### Episodic indicators

Episodic indicators include event-based impacts and are mainly derived from hydrodynamic modeling of the estuary [52]. We used the aforementioned hydrodynamic model for Barnegat Bay, implemented for Hurricane Sandy [53], to characterize the change in sediment supply, salinity, and salinity exposure. Given the infrequent occurrence of events such as Hurricane Sandy, this analysis is used as an example of how chronic and episodic forcings can be considered in tandem.

- **Lower sediment supply (SEDIM)**: During storm surges the flow over the marsh loses its momentum, thereby allowing sediment to deposit. Fine sediment is also trapped by adhering to the marsh vegetation. For this reason, the modeled change in SSC during Hurricane Sandy [53] has been used as an indicator of the geospatial variation of sediment supply to each marsh unit.
- **Larger change in salinity (SALIN) and longer salinity exposure (EXSAL)**: The change in the maximum salinity and the maximum value of the salinity exposure were calculated by the Hurricane Sandy simulation [53] and summarized over the marsh units.
- **Marsh condition change (CONDC)**: Marsh condition change and surge persistence due to Hurricane Sandy has been assessed using radar and optical data collected before and after the storm [54]. The marsh condition change rankings were averaged within each marsh unit to provide this layer.
- **Exposure potential to environmental health stressors (SCORR)**: Exposure potential to environmental health stressors was calculated with the Sediment-bound Contaminant Resiliency and Response (SCoRR) ranking system [55]. SCoRR is a ranking system based on potential sources of contamination as denoted by the U.S. Environmental Protection Agency’s Toxic Release Inventory and Facility Registry Service, related literature [56], and an expert review panel. SCoRR values were calculated at a 0.0003° (∼30 m) resolution grid and averaged over each conceptual salt marsh unit to create this layer [57].

### Ranking and wetland vulnerability index formulation

We ranked values in each dataset to indicate relative vulnerability, with four categories: low, moderate, high, and severe. (Table 1). The thresholds for each indicator were determined with a percentile classification method to maintain the same number of values in each category. The original values in each dataset were reorganized prior to categorizing so that a higher rank indicates higher vulnerability, e.g., lower tidal range is assigned a higher rank, indicating higher vulnerability. Ranking allowed for values in each dataset to be categorized with a similar distribution, ensuring a consistent definition of relative vulnerability across different layers within the study area.

**Table 1:** Vulnerability indicators. Thresholds for each indicator were determined by percentile classification for each data layer. The original values in each dataset were reorganized for higher rank to indicate higher vulnerability.

Label	Indicator	Units	Rank			
			Low	Moderate	High	Severe
CONDC	Marsh condition change	–	0.50–1.50	1.50–2.50	2.50–3.50	3.50–4
SCORR	More contaminant prone	–	0–2.35	2.35–4.50	4.50–6.15	6.15–39.62
ELEVA	Lower elevation	*m*	0.60–3.45	0.44–0.60	0.31–0.44	0–0.31
EXSAL	Longer salinity exposure	*g kg*^−1^ *d*	0.25–56.44	56.44–69.87	69.87–82.18	82.18–114.95
RESID	Higher residence time	d	0.03–0.32	0.32–0.75	0.75–10.42	10.42–50.63
SALIN	Higher salinity change	*g kg*^−1^	0.29–10.99	10.99–16.63	16.63–25.80	25.80–32.69
SEDIM	Lower sediment supply	*kg m*^−3^	0.66–0.77	0.62–0.66	0.49–0.62	0–0.49
SHORE	Shoreline change	*m y*^−1^	[-0.20 .. 9.48]	-0.40–-0.20	-0.74–-0.20	-0.74–-9.29
TIDER	Lower tidal range	*m*	1.02–1.13	0.29–1.02	0.20–0.29	0–0.20
UVVR	More unvegetated	–	0–0.04	0.04–0.11	0.11–0.25	0.25–4.05

We defined the wetland vulnerability index (WVI) as the arithmetic mean of the ranked values (Eq 1),

$$\begin{array}{c} WVI=\frac{I_{1}+I_{2}+I_{3}+...+I_{N}}{N} \end{array}$$ (1)

where *I*_*i*_ is the ranked indicator for data layer *i* and *N* is the total number of data layers. Similarly, separate wetland vulnerability indexes were defined considering only chronic or only episodic indicators (WVIC and WVIE, respectively).

### Principal Component Analysis (PCA)

To compensate for limitations of using a single aggregated index, researchers discussed the application of Principal Component Analysis (PCA) to group co-varying indicators into orthogonal components for socio-ecological studies [26, 27, 58, 59]. Through an analysis of variance, such as PCA, the contributions that share the same spatial variability are isolated into certain principal components and their contribution to the index adequately weighted. The PCA method, described in detail by [60] and [15] is a statistical tool to transform data from a n-dimensional variable space to a smaller sub-space of reduced dimensions. The expectation in PCA analysis is that the first few components account for the majority of the variance in the dataset. There are numerous methods to select the number of PCs to retain [61]. We have considered three methods, a scree plot of eigenvalues that displays a deflection point, which can be used in the selection of PCs; selecting PCs with the largest eigenvalues until a threshold percentage is achieved (> 85%); and selecting PCs with eigenvalues larger than the average of eigenvalues. PCA also allows for relationships between different variables to emerge from the analysis. This implies that for a single indicator, one can have a positive or negative value associated with the observed variables affecting a specific location. This provides information of the interaction among different variables affecting a given location.

### Cluster analysis

Cluster analysis, a technique used in machine learning algorithms [62], groups data points that share certain characteristics and are distinct from other points in the parameter space. After the grouping, an analysis of variance for each of the clusters that share distinct features can be performed. Then, the main PCs of each cluster can be combined into a global index. A potential benefit of the approach is that it allows for a simpler interpretation, as marsh units with similar characteristics are grouped together, while maintaining the main features of the variability within a global index. Several approaches are available for cluster separation (e.g., k-means, c-means) and in the present study we used Expectation-Maximization (EM) to estimate the parameters that characterize each of the clusters. The EM algorithm [63] finds the best Gaussian Mixture Model describing the data distribution. In previous studies, EM was used to estimate missing values for oceanographic datasets [64, 65].

In the present study, we first identified individual subdomains by applying cluster analysis to the ranked indicators. We first determined the number of clusters, component distributions (each with a mean and a covariance), and their respective likelihoods, and then conducted the PCA analysis on the covariances to analyze the variability within each cluster.

To determine the number of clusters, we chose the empirical Bayesian Information Criterion (BIC) [66], which identifies the number of component distributions in the data. In general, the goodness of fit improves as the number of clusters is increased. BIC optimizes goodness of fit while including a term to penalize overfitting that increases with increasing number of clusters.

### Hotspots and contributing variable classification

We identified vulnerability hotspots based on individual marsh units with a vulnerability index above a threshold value that corresponded to the mean plus one standard deviation (WVI > 0.625). We defined potential vulnerability hotspots in the vicinity of these units based on proximity (marsh units with their centroids within 1 *km* distance from the vulnerable unit), and a requirement of more than one unit within that distance (n > 1) with a threshold surface area (area ≥ 2 *km*^2^). The distance and surface area criteria were imposed to limit the number of zones to be considered in spatial trend analysis. Specifically, we created a 1 *km* buffer around the marsh units that exceed the vulnerability threshold. Then the marsh units within each buffer polygon were grouped to create the zones and the minimum area and minimum number of marsh units requirements were imposed to define the vulnerability zones.

## Results

### Spatial variation of indicators

Some of the indicators had steady gradients along the bay while others had less organized trends Fig 2. Considering the persistent indicators, there was a general longitudinal gradient in elevation, with higher elevation marshes in the south, and lower elevation marshes in the north. Similarly, tidal range in the study area attenuated gradually from more than a meter at Little Egg Inlet to less than 0.2 m in the north. Residence time also had a strong longitudinal gradient with longest values in the northern Barnegat Bay and shortest values in Great Bay, a trend mainly influenced by the size of each inlet and connectivity of the bay with the ocean. The shoreline change values were less spatially organized (positive for accretion and negative for erosion). The largest accretion rates occurred at the southward migrating tip of the southern barrier island (north of Little Egg Inlet), whereas the largest erosion rates were along the bay-side shorelines of the back-barrier marshes in Great Bay. UVVR was generally larger in the marshes in Barnegat Bay than in Little Egg Harbor and Great Bay. On the contrary the largest UVVR was also in Great Bay; in two managed open-water areas that were disconnected from normal tidal flows.

In terms of episodic indicators, sediment supply was largest in northern Barnegat Bay due to lower elevation and stronger wind-wave resuspension. Additionally, sediment supply was expectedly larger at estuary-adjacent marsh units as opposed to inland units. The model results indicated that storm surge caused the largest salinity changes to occur at inland marshes. The change in salinity exposure was smaller in northern Barnegat Bay, because of the increased freshwater input through Toms River and the setdown induced by northerly hurricane winds during the initial phase of the storm. In terms of marsh condition change previous research [54] found the change to be correlated with surge persistence, with inland marshes appearing more resilient due to fresh water discharge.

### Wetland vulnerability index

A cross-correlation analysis of the vulnerability indicators (Table 2) showed that the largest correlation was between the vulnerability to tidal range (TIDER) and vulnerability to residence time (RESID). This was consistent with earlier findings [51] that showed a strong along-estuary gradient in residence time and tidal range. The correlation between vulnerability to elevation (ELEVA) and TIDER was consistent with studies that show increased belowground biomass production and vertical growth with increasing tide range [20, 22]. On the other hand, vulnerability related with suspended sediment supply (SEDIM) was inversely correlated with TIDER (i.e., during Hurricane Sandy sediment supply was higher in the north opposed to lower tide range). The vulnerability to potential contamination from registered facilities was lower in the south than the north, similar to the vulnerability from tidal range. This emerged as a coincidental correlation between the vulnerability to potential contamination (SCORR) and TIDER. A significant positive correlation was between ELEVA and UVVR indicators resulting from the fact that marsh units with lower elevations had higher UVVR (i.e., larger unvegetated areas). The rest of the variables had smaller or mostly no significant correlation.

**Table 2:** Table of correlation coefficients. Correlation between the vulnerability indicators shown in Table 1. Positive indicates contribution to vulnerability in the same direction. Blue indicates correlations above 0.4. Red shows correlations lower than -0.4.

	**CONDC**	**SCORR**	**ELEVA**	**EXSAL**	**RESID**	**SALIN**	**SEDIM**	**SHORE**	**TIDER**
**CONDC**	1								
**SCORR**	-0.21	1							
**ELEVA**	-0.01	0.13	1						
**EXSAL**	0.15	-0.13	0.25	1					
**RESID**	-0.10	**0.41**	**0.43**	-0.21	1				
**SALIN**	-0.23	0.29	-0.09	0.15	0.12	1			
**SEDIM**	0.28	-0.31	-0.25	0.28	**-0.53**	-0.08	1		
**SHORE**	0.10	-0.04	0.05	-0.10	0.13	-0.07	-0.18	1	
**TIDER**	-0.14	**0.45**	**0.50**	-0.18	**0.93**	0.11	**-0.57**	0.13	1
**UVVR**	0.00	-0.11	**0.51**	0.21	0.14	-0.05	-0.08	0.15	0.20

**Fig 3:**
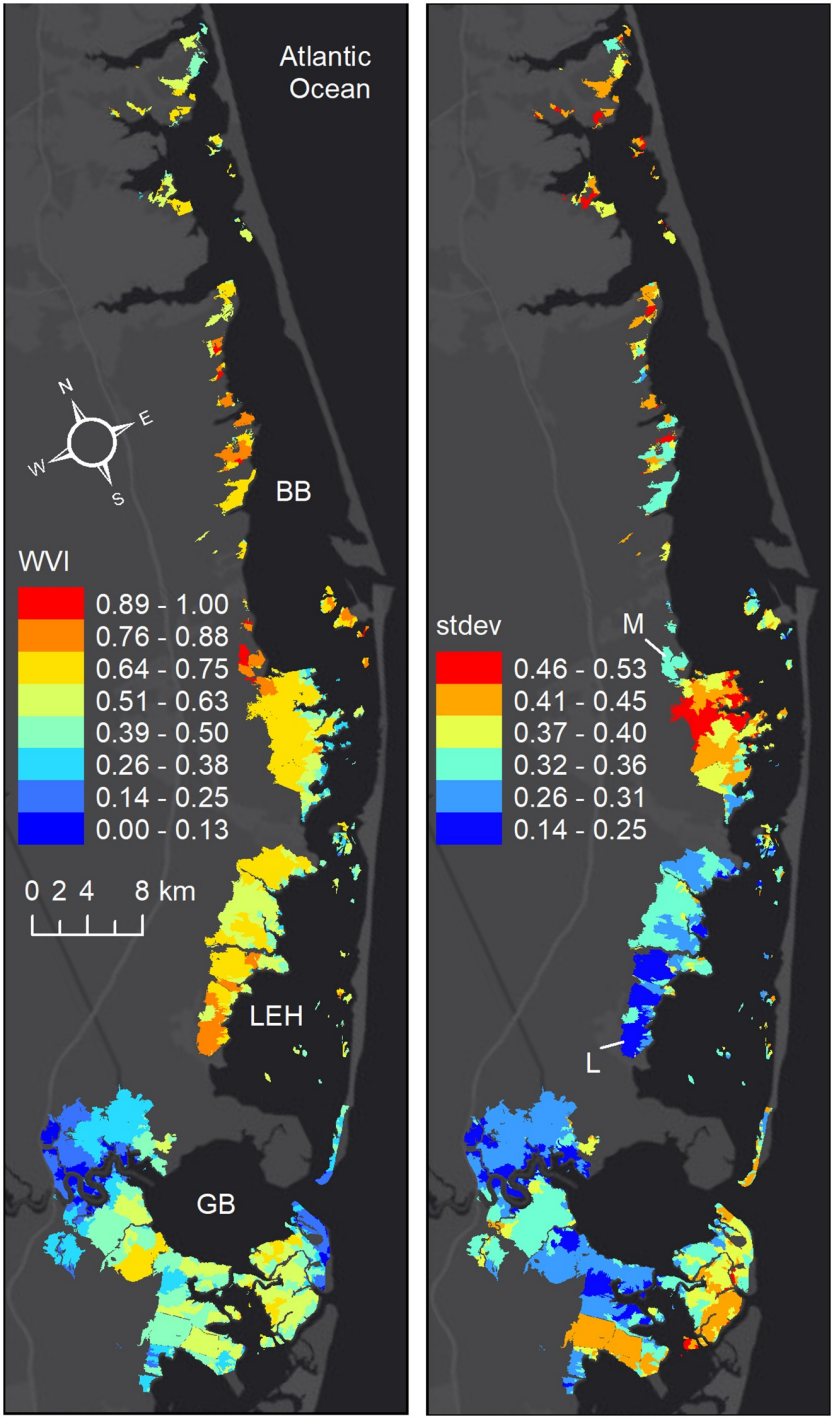
Wetland vulnerability index. a) Wetland vulnerability index (WVI) based on all of the data layers, and b) the standard deviation of parameters at each marsh unit (BB: Barnegat Bay, LEH: Little Egg Harbor, GB: Great Bay). L and M are two locations where vulnerability was severe, but contribution from indicators were more uniform at L.

The wetland vulnerability index (WVI) showed higher vulnerabilities in the northern part of the domain (Fig 3a). In general, the bay-wide average for WVI was higher in the Barnegat Bay (BB) marshes than Little Egg Harbor (LEH), which was higher than Great Bay (GB). Clusters of marsh units had the lowest vulnerability (0—0.25) in Great Bay in the south, and a series of marsh units had severe vulnerability (0.75—1) in Barnegat Bay in the north. The most severe WVI occurred in Barnegat Bay. Mapping the standard deviation of all of the vulnerability indicators at a single marsh unit provided more insight into the nature of vulnerability (Fig 3b). When the standard deviation was small the contribution from the layers were likely to be in the same direction, and they were likely to be less uniform as it increased. For example, both at the southern tip of the Little Egg Marshes (location L) and the northern tip of the Manahawkin marshes (location M) vulnerability was severe. However, the contribution from the underlying indicators to vulnerability was more uniform at location M (SHORE and EXSAL were severe and all others were high) in comparison to location L (SHORE was low, CONDC was moderate, SCORR was high and all others were severe). When the vulnerability was low or severe, standard deviation tended to be smaller by definition. In contrast, when it ranged from moderate to high the largest standard deviations existed more often (e.g., across the middle of Manahawkin marshes, scattered throughout BB, and some locations in GB).

**Fig 4:**
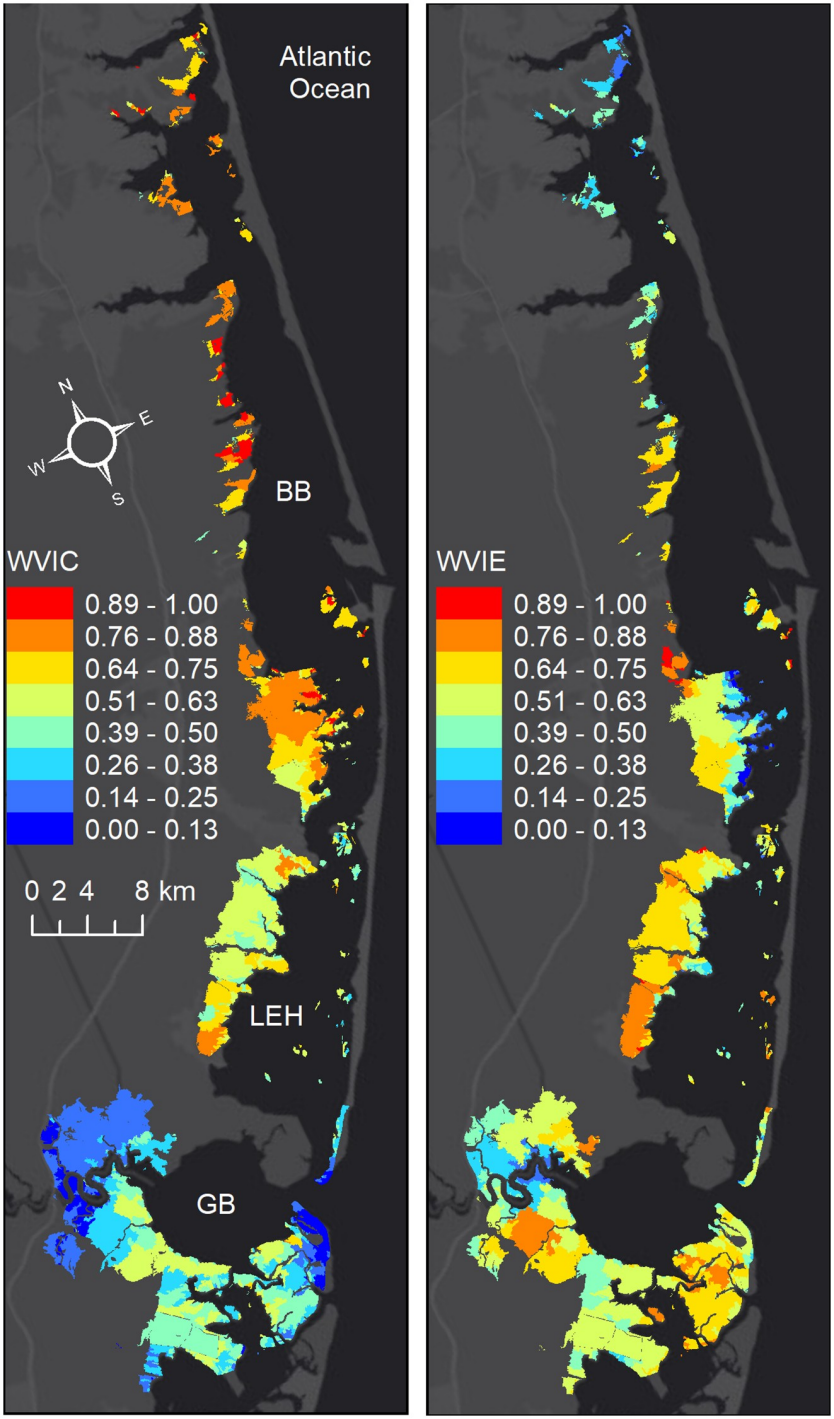
Chronic versus episodic wetland vulnerability index. a) Based on chronic indicators (WVIC; consisted of elevation, mean tidal range, shoreline change rate, UVVR, residence time vulnerability indicators), and b) based on episodic indicators (WVIE; consisted of change in sediment concentration, change in salinity and salinity exposure, marsh condition change, and exposure to contaminants vulnerability indicators).

When only the chronic indicators are considered, a similar north-south gradient can be seen between WVI and WVIC. However, larger WVIC values and lower values of WVIE in the northern part of Barnegat Bay indicated that the contribution to WVI in this area was from chronic conditions (Fig 4a). The spatial variation of marsh vulnerability in LEH, was determined by both the episodic and chronic factors (Fig 4b). The cross-shore gradient seen in WVI at the Manahawkin marshes (Fig 1) was a result of the cross-shore variation WVIE in this area. The contribution of episodic factors to vulnerability was low at the bay-side Manahawkin marsh units and in the northern Barnegat Bay. Overall the variation in marsh vulnerability was explained mainly by the chronic indicators in the north in BB, by episodic factors in the south in GB, and by a combination of both episodic and chronic indicators in LEH.

### PCA results

When PCA was applied to the entire dataset of 10 indicator variables, the resulting principal components explained a similar percentage of the total variability. The scree plot of the eigenvalues did not have a clear inflection point and at least six principal components were required to explain 85% of the variance. The variance explained by the first six components were 31%, 17%, 13%, 10%, 9% and 6% (Table 3). Only the first three PCs had eigenvalues that were above the average eigenvalue.

**Table 3:** Principal components analysis. Principle component loadings (PC1–PC10) for each vulnerability indicator. Blue indicates loadings with a magnitude greater than 0.4.

	PC1	PC2	PC3	PC4	PC5	PC6	PC7	PC8	PC9	PC10
**CONDC**	0.16	-0.30	-0.28	**0.75**	-0.09	0.24	0.15	0.38	0.07	0.03
**SCORR**	-0.32	0.29	0.24	0.31	-0.04	**-0.72**	0.26	0.27	-0.08	-0.04
**ELEVA**	-0.32	**-0.49**	0.14	-0.02	0.21	-0.18	-0.13	-0.06	**0.73**	-0.05
**EXSAL**	0.12	**-0.42**	**0.53**	0.17	-0.17	-0.12	**-0.54**	0.00	**-0.40**	-0.01
**RESID**	**-0.51**	0.00	-0.08	0.23	0.05	0.23	0.00	-0.35	-0.23	**-0.67**
**SALIN**	-0.10	0.25	**0.58**	0.05	**-0.51**	**0.44**	0.19	0.04	0.31	0.01
**SEDIM**	**0.41**	-0.14	0.17	0.24	0.03	-0.19	**0.45**	**-0.70**	0.04	0.04
**SHORE**	-0.10	-0.16	**-0.44**	-0.11	**-0.80**	-0.27	-0.08	-0.18	0.06	0.00
**TIDER**	**-0.53**	-0.03	-0.04	0.18	0.07	0.15	-0.01	-0.27	-0.20	**0.74**
**UVVR**	-0.16	**-0.55**	0.11	-0.38	-0.04	0.06	**0.60**	0.25	-0.31	-0.03
Proportion	**0.31**	**0.17**	**0.13**	**0.10**	**0.09**	**0.06**	**0.06**	**0.05**	**0.03**	**0.01**

**Fig 5:**
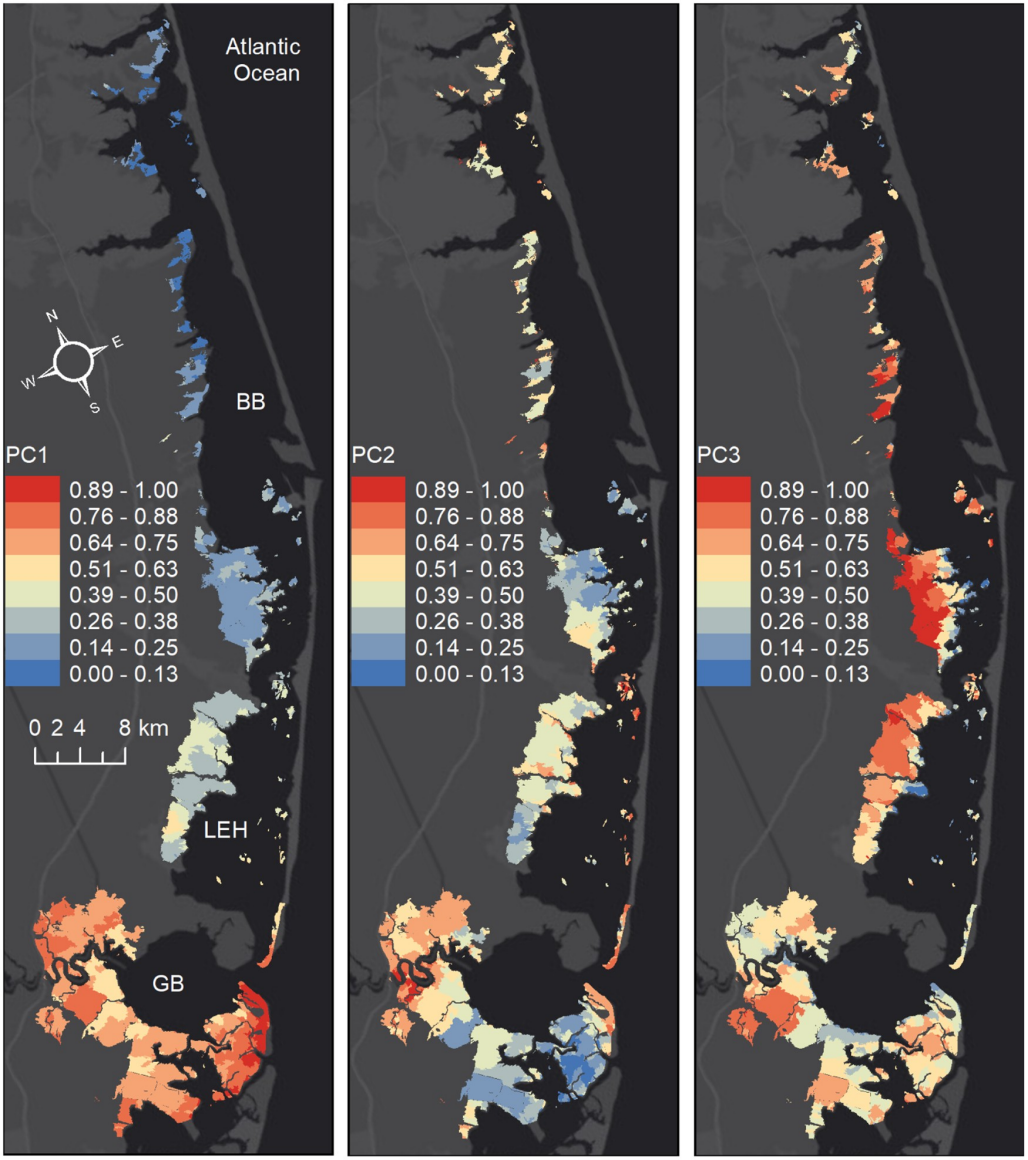
Map of principal components. Spatial distribution of the first three principal components over the entire domain.

PC1 was correlated with RESID and TIDER, two variables that also had a strong correlation (Table 2). The spatial distribution of PC1 (Fig 5a) showed a north-south gradient following the characteristics of the major PC1 contributors, RESID and TIDER (Fig 2). SEDIM also had a moderate north-south gradient, but its contribution to PC1 was opposite of RESID and TIDER. Therefore, larger values in the PC1 map highlight the lower vulnerability to RIDER and RESID, but higher vulnerability to SEDIM.

PC2 was mainly correlated with UVVR and ELEVA, which were also positively correlated among each other. The other positive correlation described with these two components was between CONDC and EXSAL. PC2 separated EXSAL from SALIN (-0.42 vs. 0.25), highlighting the marsh units where there was larger salinity variation during the hurricane, but the salinity exposure was limited by the shorter duration of inundation. Consequently, larger values of in PC2 map indicates lower vulnerability to ELAVA, UVVR and SEDIM (Fig 5b).

PC3 on the other hand, had the largest positive contributions from EXSAL and SALIN and the largest negative contribution from SHORE. Therefore, larger values of PC3 map indicates marsh units that are vulnerable to both salinity change and exposure to salinity but resilient to shoreline change. These are mainly the units that are more inland (Fig 5c). East-west gradient of EXSAL and SALIN in Fig 2 across the Manahawkin marsh can clearly be seen in PC3.

None of the variables in the first three PCs were likely to individually dominate (because of similar contribution from many variables and magnitude of maximums less than 0.6). Because PCA does not use the location information for the data and considering the low percentage of explained variance by the first three PCs, a direct decomposition of the data with PCA might not be the optimal approach. However, it is still a powerful tool discover the general trends in the underlying data. Effects of a cluster analysis on the PCA results are presented in the next section.

### Cluster analysis results

**Fig 6:**
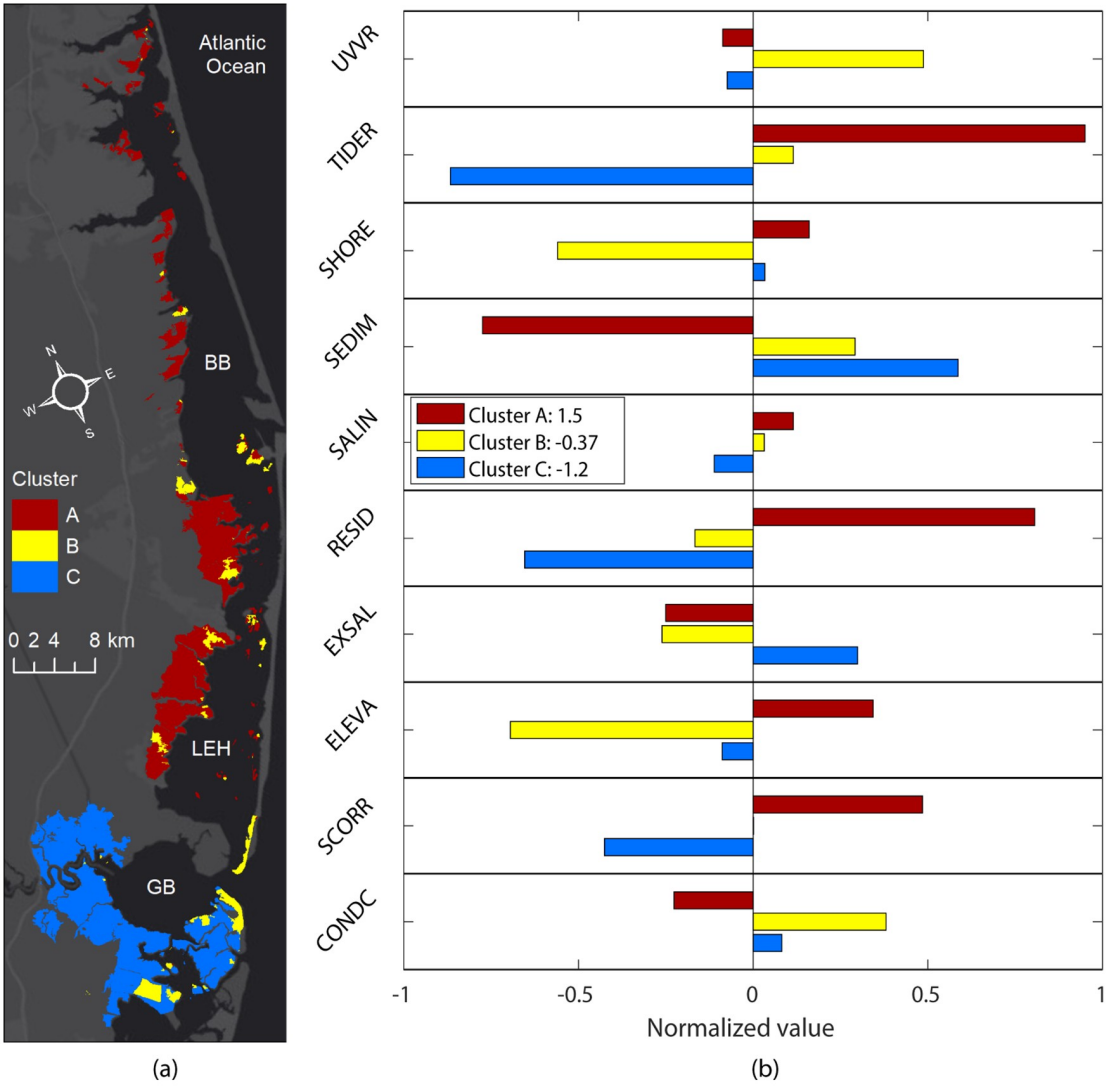
Cluster classification. a) Spatial distribution of the three clusters obtained using Expectation-Maximization to separate the sub-components. b) Departure from the mean of each indicator variable for all clusters. The values of the variables have been normalized with positive values representing cluster means higher than global average. A value of one represents a departure of one standard deviation from the global mean of that variable. The values in the legend represent the sum of all normalized contributions for each cluster.

The BIC selected for three component distributions in the dataset (Fig 6). The clustering was conducted on the normalized dataset (mean of each variable subtracted and result divided by standard deviation of that variable). The three components had distinct spatial characteristics (Fig 6a). The first cluster included most marsh units in the interior of the BBLEH system that were relatively less exposed to the influence of the inlets. The majority of the second cluster included units in the BBLEH and Great Bay systems that were influenced by the exchange through the inlets (most of them in close proximity to inlets). The third cluster included most of the marsh units inside Great Bay.

The first cluster (Cluster A, BBLEH interior) had higher than global average (mean of values from all marsh units regardless of cluster) residence time and tidal range effect on vulnerability (smaller tidal range) and lower than normal sediment contribution to vulnerability (more available sediment) (Fig 6b) and included more than 50% of the marsh units. The second component (Cluster B, inlet influence, 18% of marsh units) had higher than average condition and UVVR means and lower than the global average shoreline and elevation contributions to vulnerability (higher elevation and less shoreline erosion). The third component (Cluster C, Great Bay, 31% of marsh units) had higher than average sediment and lower than global mean values for contaminants, residence, and tidal range contributions to vulnerability.

The overall effect was that Cluster A (BBLEH interior) exhibited more vulnerable conditions (sum of all normalized variables in that cluster is 1.5), the second cluster was slightly less vulnerable (-0.37 normalized sum) than the average of the system (global average is zero), and the third cluster was even less vulnerable (-1.2 normalized sum) when compared with the entire system. This result suggested that in general the GB marsh units were less vulnerable (e.g., smaller WVI) than the BB units. We also determined the variables that contributed more to the differentiation between regimes. The variables that contributed more to the cluster separation were elevation, residence time, sediment supply, and tidal range. Salinity was the variable that contributed least to the separation.

**Fig 7:**
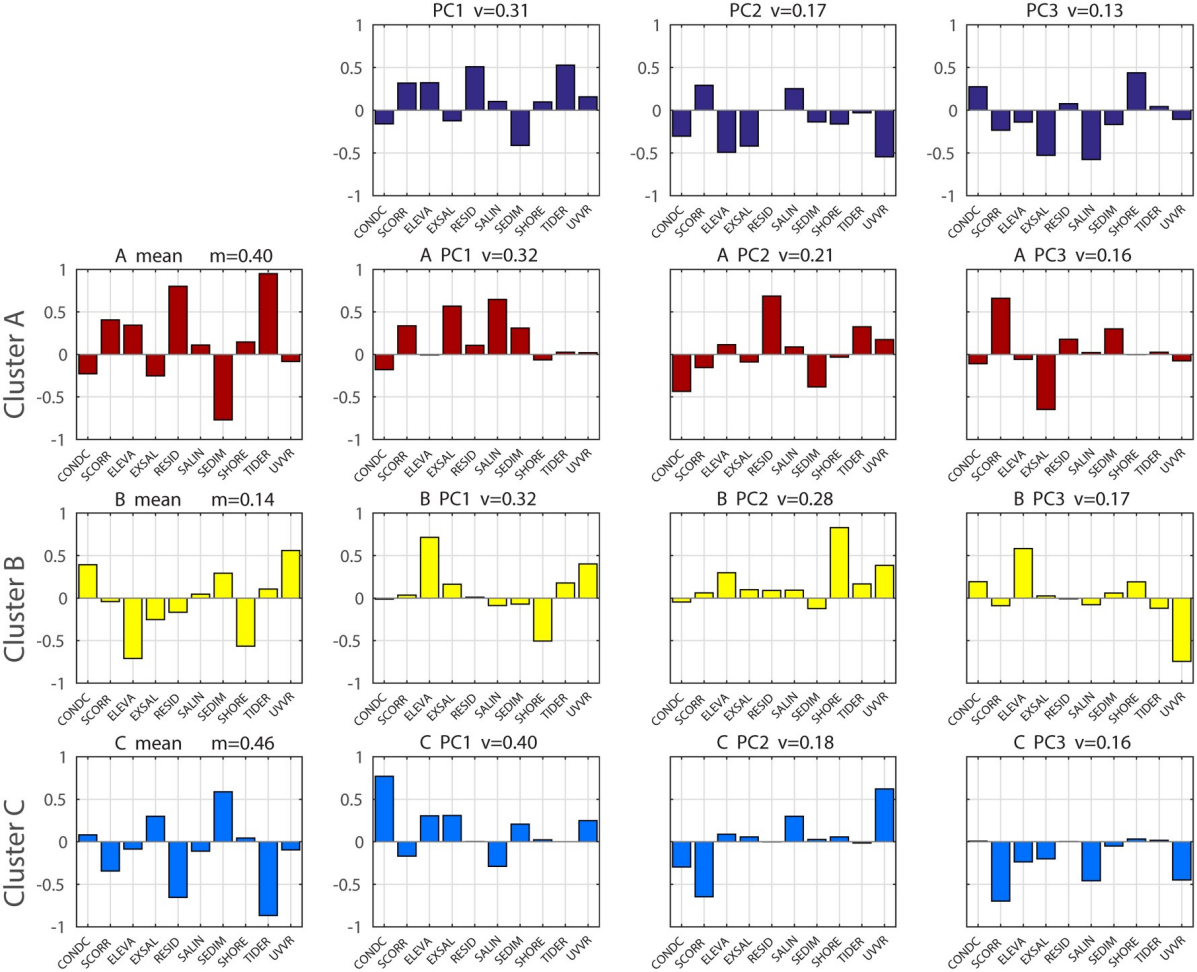
PCA analysis of the entire dataset and the three different clusters obtained using Expectation-Maximization to separate the components. First row: PCs of the entire dataset without cluster separation. Second row: Means and PCs of Cluster A (BBLEH interior). Third row: Means and PCs of Cluster B (inlet influence). Fourth row: Means and PCs of Cluster C (Great Bay). *v* is the fraction of total variance explained by that PC component. *m* is fraction of total marsh units included in each cluster.

In general, the benefit of PCA after clustering is that it characterizes variance in groups that share common features. When applied separately to each cluster, PCA resulted in a slightly better decomposition of components than then applied globally to the entire dataset (Fig 7). The variation explained by the first three components at each cluster was slightly larger than with the global PCA (69%, 77%, and 74%, respectively, versus 61% globally). Each cluster can be individually characterize further following the approach from the global PCA. For instance, the first PC of Cluster A (BBLEH interior) was related to exposure and salinity variability (32% of variance), while the first PC of Cluster B (inlet influence) was associated with variability in elevation and UVVR (32% of variance), and finally the first PC of Cluster C (Great Bay) was related to marsh condition variability (41% of variance). The number of PCs required and the amount of variability explained in both the global and clustered PCA were similar, which supported the earlier assumption of equal weight contributions to WVI from all indicators.

### Transect analysis results

**Fig 8:**
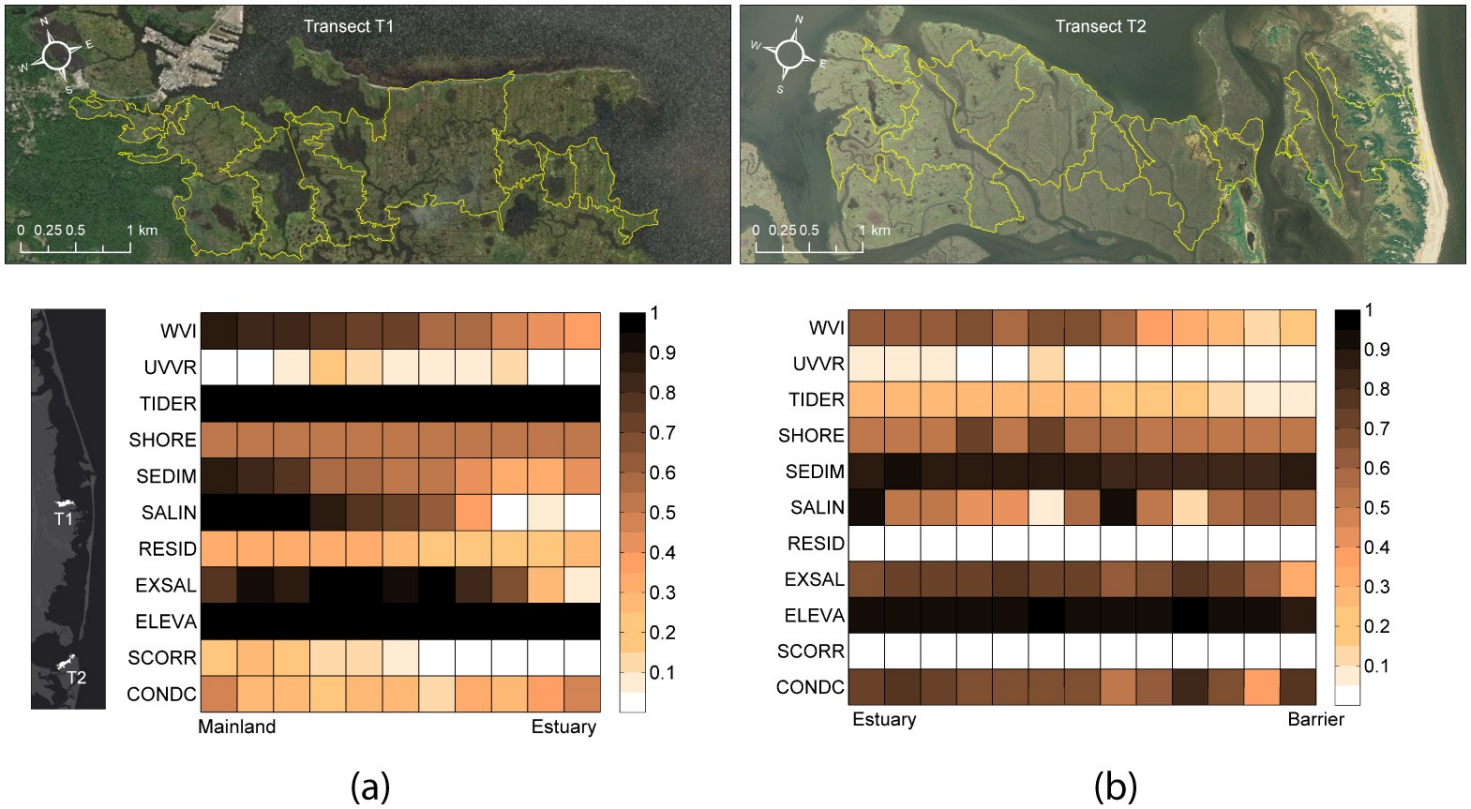
Change in underlying parameters. a) along a west-east transect T1 over the Manahawkin marsh; and b) along a transect T2 from the barrier to the estuary in Great Bay. The values are in terms of WVI in the entire estuary. The transect locations are indicated in the adjacent map.

An east-west cross-section (transect T1) across the marsh units at Manahawkin Marshes (Fig 8a) showed a uniformly decreasing WVI pattern moving from mainland towards estuary. The WVI pattern was a result of decreasing contributions from multiple variables in the same direction, including mainly the vulnerability related with less sediment supply (SEDIM), change in salinity (SALIN) and potential of exposure to contamination (SCORR). Some of the other variables also contributed to vulnerability but remain close to constant in the transect: vulnerability to low tide range (TIDER) and low elevation (ELEVA) were severe (close to one) at every marsh unit along transect T1 and vulnerability to shoreline change (SHORE) was constantly moderate.

Another fairly uniform gradient in WVI was along transect T2 across the back-barrier marshes in Great Bay (Fig 8b), but with decreasing vulnerability going from estuary towards barrier island. This was because of a similar trend in TIDER, UVVR and CONDC, but was also a combination of different indicators. The contributions from RESID and CONDC were negligible (close to 0) for the marsh units along transect T2. When the two transects were compared, the pattern of vulnerability increased from marsh units near the bay toward inland units in the BBLEH system, whereas in GB the pattern was reversed with less vulnerable units being farther from the bay towards the barrier island.

### Hotspot results

**Fig 9:**
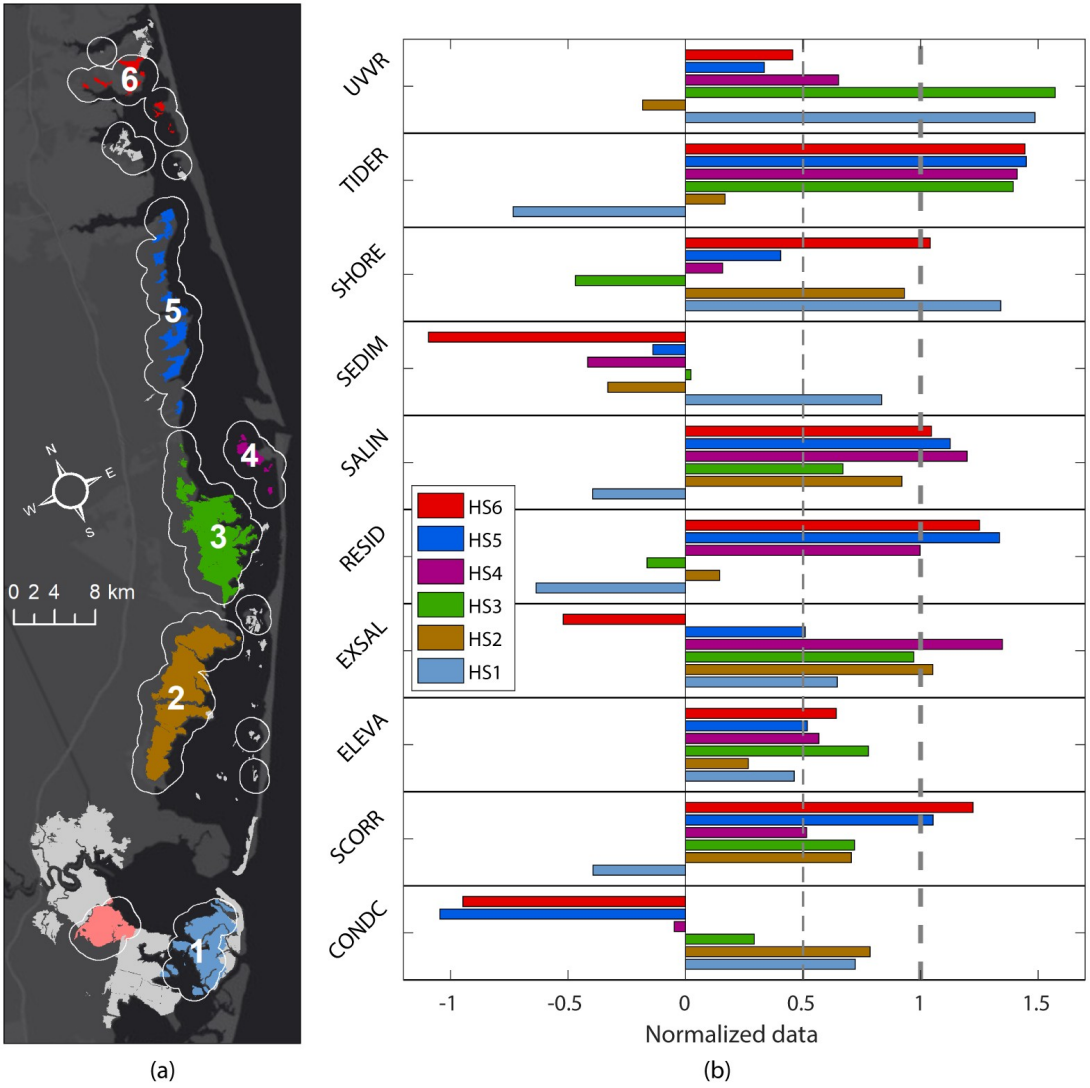
Vulnerability hotspot zones and trend analysis. a) The zones that satisfy both the minimum area and the minimum number of marsh units are marked as vulnerability hotspots and assigned a number. Lines indicate the 1 *km* buffer used to define the zones. The zones that satisfy only the area criterion are shown in color other than light gray. b) Contribution of each normalized parameter to vulnerability in each of the hotspot zones. Positive values represent above average contributions to vulnerability with values exceeding 1 (one standard deviation from average) being significantly different from the global mean.

Based on our definition of vulnerability hotspots (WVI > 0.625 with *n* > 1 within 1 *km*, and area ≥ 2 *km*^2^), we identified six vulnerability hotspots (Fig 9a). The total area (and the number of marsh units) for zones one to six was 14.6 *km*^2^ (173), 28.6 *km*^2^ (125), 23.6 *km*^2^ (158), 7.7 *km*^2^ (95), and 2.6 *km*^2^ (45), respectively. Grouping together marsh units in hotspot zones allowed for identifying vulnerability characteristics that may be common for a specific area.

The contribution of each of the variables to each hotspot zone showed distinct differences between areas. Hotspot HS1 in GB exhibited significantly higher than average shoreline change and UVVR vulnerability (SHORE and UVVR > 1 in Fig 9b). Hotspot HS2 at mainland marshes in LEH had higher than average EXSAL with smaller contributions SALIN and SHORE. Hotspot HS3 (Manahawkin marsh) exhibited the largest normalized contribution to vulnerability and it was caused by UVVR. TIDER and EXSAL were also contributing significantly to the vulnerability of this hotspot. The marshes near Barnegat Inlet (hotspot HS4) were more influenced by TIDER, EXSAL, SALIN, and RESID. Hotspot HS5 (mainland marshes in BB) exhibited high vulnerabilities associated mainly with TIDER, RESID, SALIN and SCORR. Finally, the vulnerability of Hotspot HS6 (northern marshes) was linked to the same factors (TIDER, RESID, SALIN and SCORR) with the additional contribution of shoreline change.

Several factors (TIDER, RESID, SALIN, EXSAL) exhibited higher than average contributions to high vulnerability in multiple (3-4) hotspots. Meanwhile, CONDC, ELEVA and SEDIM were not significantly different than the mean in any hotspot. This fact did not imply that they were not contributing to marsh vulnerability, but rather that their contributions were either constant across all hotspots (e.g., ELEVA) or that some marsh units in each of the hotspots contributed to lower the overall pattern of that variable in that hotspot (e.g., SEDIM).

## Discussion

In this section we discuss the results of our data synthesis to assess the vulnerability of EBFNWR salt marsh complex by looking into pairwise relations between the underlying parameters, and presenting the advantages and relevance of the study to coastal wetland management.

### Pair correlation

**Fig 10:**
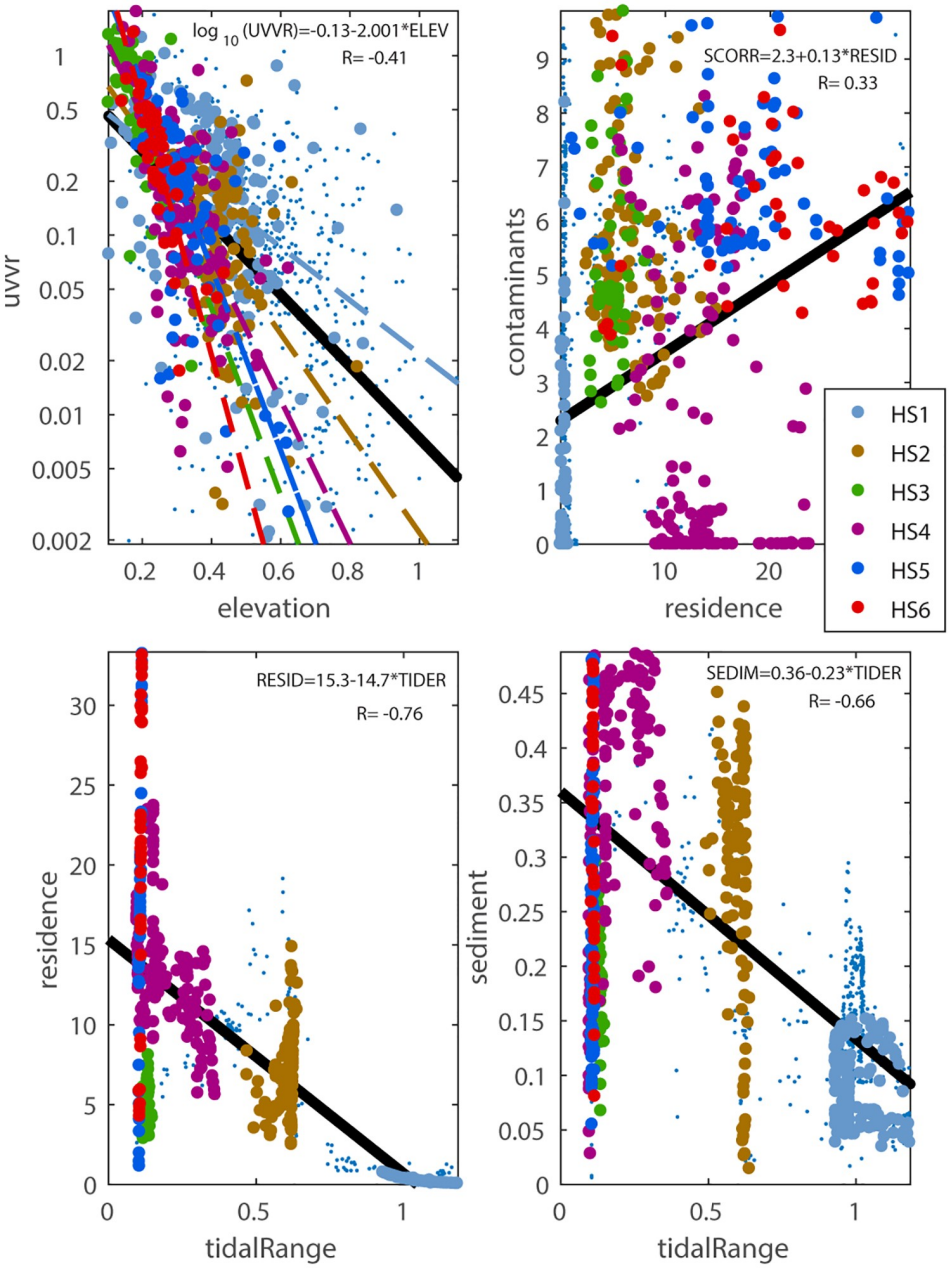
Relation between pairs of variables with significant (P < 0.001) correlations. a) UVVR vs elevation; b) Contaminants vs residence time; c) residence time vs tidal range; and d) sediment vs tidal range. The data points are color coded by each hotspot (see Fig 9 for hotspot location). The black line is the linear regression fit for each pair with equation and coefficient of determination indicated. For UVVR vs elevation individual regression lines for each hotspot are also displayed color coded.

Certain pairs of parameters exhibited significant correlations (Fig 10). For instance, UVVR and elevation relation followed an exponential decay function (*R* = −0.41, *p* < 0.001, Fig 10a), with low elevations being related to high values of UVVR. The rates of exponential decay function for most of the hotspot regions (except hotspot HS1) were larger than the global relationship, indicating that highly vulnerable areas (hotspots) tended to be associated with higher UVVR values than normal marshes at low elevations.

There was a significant positive correlation (*R* = 0.33, *p* < 0.001) between residence time and contaminants (Fig 10b) that explained around 10% of variance. Most vulnerability hotspot areas tended to have contaminant values above the global linear relationship. One exception was hotspot HS4 that included marshes along the barrier island near Barnegat Inlet, which was relatively far from any of the contaminant point sources. This can be attributed to having more registered facilities and encroachment of marshes by development towards the northern part of the domain. The resulting higher vulnerability to contaminants showed similarity to the vulnerability to low tidal range in the north.

Tidal range and sediment availability exhibited a significant negative correlation (*R* = −0.66, *p* < 0.001; Fig 10c). Areas of large tidal range in the south (e.g., hotspot HS1 in Great Bay) had less sediment, while areas in the northern part of Barnegat Bay had larger sediment concentration during the storm as sediment was transported from south to north.

Tidal range and residence time were correlated (*R* = −0.76, *p* < 0.001) for this domain (Fig 10c) but the correlation was not high enough to exclude one of them from the vulnerability index. In fact, one variable explains less than 60% of the variance from the other, so excluding one would have removed substantial and, in some areas maybe critical, information. Additionally, in other estuaries where stronger riverine discharge enhances flushing, residence time and tidal range may not be highly correlated. For these reasons, we included both of these parameters for contrasting to prospective applications of the method to other estuaries.

### Spatial footprint

Coastal wetlands usually extend continuously over large areas, which makes it difficult to plan for systematic management. Our physics based delineation of the marsh system into coastal marsh units provides flexibility. Dividing a large complex into smaller units reveals spatial variation of physical state within a complex and facilitates prioritizing parts of it for action.

Field measurements are an essential part of wetlands research. Here we have used results from numerical models calibrated with field measurements and supplemented them with remote sensing measurements where appropriate. Spatial coverage is scattered when only a limited number of point measurements can be provided by field campaigns. In comparison, as we demonstrated here, aerial imagery and remote sensing data provided seamless geospatial coverage over an entire domain. We combined geospatial data with image analysis methods to provide interpretive results that are consistent and complete over the entire domain. There are many studies (e.g., [11, 67–69]) that rely on point measurements (e.g., surface-elevation tables and marker horizons) and metrics defined in the vertical direction to assess the resilience of marshes at locations such as the National Estuarine Research Reserve System (NERRS; e.g., Great Bay National Estuarine Research Reserve) or Long Term Ecological Research program (LTER; e.g., Plum Island Estuary) monitoring and research sites. These vertical metrics and our geospatial approach should complement each other when used in tandem. Additionally, synthesizing various datasets enabled investigating the correlation between multiple factors and assess their cumulative impact.

### Relevance of vulnerability index

The vulnerability index approach provides a way to combine the sensitivity of the marsh system to external and internal forcing (susceptibility) and the ability to adapt to changing environmental conditions (resiliency). While the index should not be used as an indication of specific changes in the marsh system, it provides a method to highlight areas that are more likely to suffer change. By examining the contributions of different layers to the index at specific locations, it can also help identify the main factors leading to marsh changes. In most cases the socioeconomic cost of restoration may be lower at sites with a relatively small number of severe problems than at sites with less severe but many problems [70]. With this approach coastal mangers can explore the marsh system to quickly locate the most severely vulnerable units and then strategize how to allocate resources to tackle the major drivers by examining the contribution from underlying datasets. We have demonstrated this with the hotspot analysis. We selected marsh units with high vulnerability and analyze the indicators to discover local relationships among the indicators. In a case where the resources are already allocated for action against certain drivers, then the same approach can also be used to identify locations where the best return on investment is likely (i.e., prioritize shoreline protection for a unit with less vulnerability to the rest of the drivers rather than for a unit that is already severely impacted by them).

There are many challenges in designing a wetland vulnerability index: having heterogeneous datasets which may describe diverse physics and may involve units of different scales and values with unbounded limits; having parameters with unknown or yet to be established critical values; having a combination of datasets that are of episodic and chronic nature; having unclear relative weights of each parameter, etc. Many of these require assumptions to be made starting with the intermediate steps. Additionally, the method should be applicable to different domains and should be able to accommodate the technological progresses in data quality and data processing. Because of these reasons, a method that is flexible, reproducible and that can support a recurrent re-synthesis and re-analysis approach is ideal. In this study we have provided a framework demonstrating how this can be achieved with the existing datasets and our current understanding of their role in wetland vulnerability. This framework should be used in the exploratory sense and updated when a re-synthesis and re-analysis is necessary to accommodate technological changes, changes to the domain and changes in our understanding of the problem. As suggested in an earlier study [24], the imperative we have should be to apply the best possible solution while revising the methods for possible improvements instead of waiting for the perfect solution in the face of escalating impacts.

### Link to management

Aggregating all the related indicators into a single vulnerability index has the benefit of prioritizing the most critical marsh units in a systematic way. Coastal wetlands provide a broad range of ecosystem services ranging from providing natural habitat to sequestering carbon and any salt marsh loss means reduction in the capacity of these services. Loss can be caused by any combination of marsh shoreline change, exposure to contaminants, limited nutrient and sediment supply, etc. The benefit of aggregating indicators into a single vulnerability index is that it immediately reflects the overall state of the system rather than each individual indicator separately. The method we present here satisfies this condition, but also provides the underlying data and interrelations for further interpretation. Additionally, using a ranking system facilitates assessing the relative vulnerability within each dataset. Without the prior knowledge of absolute vulnerability, the parameters can be classified from low to severe based on their relative vulnerability. This makes the method consistent and applicable to any study area. A percentile ranking provides equal number of members in each bin, which assigns the same weight to each bin.

In cases with limited resources, planners can decide which marsh units to prioritize based on the ecosystems value of the marsh unit and the level of vulnerability. For example, if a coastal manager is interested in the habitat suitability for coastal waterbirds in the Forsythe complex, they can estimate the physical evolution of these habitats using the vulnerability index. They can further investigate the underlying datasets to see if there is any general, spatially uniform pattern in any specific indicators (e.g., shoreline retreat everywhere) or any severe vulnerability because of contributions from several factors locally (e.g., vulnerability to exposure to contaminants, lack of sediment supply, etc). This sort of analysis should be beneficial to identify the type of vulnerability and allocate resources accordingly early in the project lifetime.

## Conclusion

Marsh vulnerability is often not determined by a single indicator. A combination of several indicators is needed to properly assess the state of a marsh and its ability to recover from disturbances. We considered several data layers to provide information to researchers and coastal managers to evaluate wetland vulnerability. To standardize the steps taken in creating an index, we proposed a reproducible method that can also be applied to other domains. First, we introduced a novel approach to divide a marsh complex in to hydrologically defined marsh units, which facilitated assessing the parts of a large complex for relative vulnerability while reducing the subjectivity in definition of each unit. Then, we considered variables from various sources ranging from observational data to numerical models and ranked them to obtain spatially varying vulnerability indicators. The synthesis of data was done through an aggregation of the indicators to obtain a wetland vulnerability index that is customizable and extensible. We demonstrated the customization by creating two different vulnerability indices for chronic and episodic indicators separately. The extensibility comes with inclusion of new indicators.

Our analysis highlighted the strong correlation between the residence time and tide range in the E.B. Forsythe NWR. It also indicated correlation between elevation and unvegetated to vegetated marsh ratio. Sediment budgets of microtidal marsh complexes on the Atlantic and Pacific coasts of the United States consistently scale with unvegetated to vegetated marsh ratio despite differences in sea-level rise, tidal range, elevation, vegetation, and stressors [16]. This highlights UVVR as one of the strongest indicators of microtidal marsh stability. Because of its correlation with UVVR we could also conclude that the vulnerability to low elevation was a major factor that determined the regional structure in the Forsythe complex. Overall, the wetland vulnerability was highest in Barnegat Bay, and decreased slightly in Little Egg Harbor and more substantially in Great Bay. This pattern was also seen in the vulnerability index based only on the chronic indicators, while the index based on episodic indicators had a structure opposite of the chronic index in northern Barnegat Bay and Great Bay.

We provide a comprehensive set of datasets that can be used for various approaches to determine the vulnerability of coastal wetlands. The set of data from this study can be combined with other prospective studies (specifically the ones that investigate the biogeochemical aspects, such as invasive species, herbivore access, increased carbon dioxide concentration etc) to establish alternative or more comprehensive indicators. At the estuary scale, we used the marsh unit elevation as an indicator of vulnerability to sea-level rise. When applied at larger scales the spatial variability of climate change, sea-level rise become important and these can be incorporated as additional layers to construct a global index. Therefore, the index approach described in this study can be conducted in almost any spatial scale and with data layers representative of specific regional marsh characteristics. As such, it represents a flexible methodology to assess vulnerability at multiple scales and, when used properly, it can facilitate marsh-related planning and decision-making.
